# Kruppel-like factor 2 suppresses mammary carcinoma growth by regulating retinoic acid signaling

**DOI:** 10.18632/oncotarget.5767

**Published:** 2015-09-21

**Authors:** Wei Zhang, Liraz Levi, Pallab Banerjee, Mukesh Jain, Noa Noy

**Affiliations:** ^1^ Department of Cellular & Molecular Medicine, Lerner Research Institute, Cleveland Clinic Foundation, Cleveland, Ohio, USA; ^2^ Dana-Farber Cancer Institute, Boston, Massachusetts, USA; ^3^ The Case Cardiovascular Research Institute, Case Western Reserve University School of Medicine, Cleveland, Ohio, USA; ^4^ Department of Nutrition, Case Western Reserve University School of Medicine, Cleveland, Ohio, USA

**Keywords:** Kruppel-like factor, nuclear receptors, retinoic acid, vitamin A, breast cancer

## Abstract

The transcription factor Kruppel-like factor 2 (KLF2) displays anticarcinogenic activities but the mechanism that underlies this activity is unknown. We show here that KLF2 is markedly downregulated in human breast cancers and that its expression positively correlates with breast cancer patient survival. We show further that KLF2 suppresses tumor development by controlling the transcriptional activity of the vitamin A metabolite retinoic acid (RA). RA regulates gene transcription by activating two types of nuclear receptors: RA receptors (RARs), which inhibit tumor development, and peroxisome proliferator-activated receptor β/δ (PPARβ/δ), which promotes tumorigenesis. The partitioning of RA between these receptors is regulated by two carrier proteins: cellular retinoic acid-binding protein 2 (CRABP2), which delivers RA to RARs, and fatty acid-binding protein 5 (FABP5), which shuttles ligands to PPARβ/δ. We show that KLF2 induces the expression of CRABP2 and RARγ and inhibits the expression FABP5 and PPARβ/δ thereby shifting RA signaling from the pro-carcinogenic FABP5/PPARβ/δ to the growth-suppressing CRABP2/RAR path. The data thus reveal that KLF2 suppresses tumor growth by controlling the transcriptional activities of RA.

## INTRODUCTION

Members of the Kruppel-like factor (KLF) family of transcription factors bind to GC-rich sequences in promoter regions of target genes, and they either activate or repress transcription in a cell- and promoter-dependent manner [1, 2]. Several KLFs have been implicated in involvement in cancer cell biology. For example, it was reported that KLF5 promotes proliferation of breast and prostate cancer cells [3, 4] and that KLF11, KLF6, and KLF4 suppress the growth of various carcinomas [5-8]. Another member of the family that was shown to display anti-carcinogenic activities is KLF2. It was reported that KLF2 expression is downregulated in prostate and ovarian cancers [9, 10]. It was also reported that the protein suppresses the growth of T-cell leukemia cells [11], sensitizes ovarian carcinoma cells to DNA damage-induced apoptosis [10], and displays anti-angiogenesis activities [12]. The mechanism(s) by which KLF2 regulates the growth and oncogenic properties of carcinoma cells remain incompletely understood but it was reported that the factor inhibits the expression of epidermal growth factor receptor [13] and that it suppresses KRAS-induced oncogenic transformation [2]. Interestingly, as described below, recent reports suggested that there exists cross-talk between KLF2 and transcriptional signaling by the vitamin A metabolite retinoic acid (RA) [13, 14].

RA regulates transcription by activating several nuclear hormone receptors: the classical RA receptors RARs [15, 16], and peroxisome proliferator activated receptor β/δ (PPARβ/δ) [17, 18]. The partitioning of RA between these receptors is controlled by cellular RA-binding protein 2 (CRABP2), which shuttles it to RARs, and fatty acid binding protein 5 (FABP5), which delivers it to PPARβ/δ. CRABP2 and FABP5 are cytosolic in the absence of their ligand, but they move to the nucleus upon binding of an activating ligand such as RA [17, 19-22]. In the nucleus, these binding proteins selectively ‘channel’ RA to their cognate receptors and thus markedly enhance their transcriptional activities [17, 20, 22-24]. Consequently, RA activates RARs in cells that express a high CRABP2/FABP5 ratio, but can function through PPARβ/δ when this ratio is low. As RAR and PPARβ/δ regulate the expression of distinct cohorts of genes, RA displays different biological activities in cells where it activates RAR, and in cells in which it functions through PPARβ/δ. For example, in many carcinoma cells, RAR upregulates genes that trigger differentiation, apoptosis and cell cycle arrest [25-32] while PPARβ/δ induces the expression of genes that promote proliferation, angiogenesis and survival [17, 33-37]. Consequently, RA inhibits the growth of carcinoma cells in which the CRABP2/FABP5 ratio is high such as MCF-7 mammary carcinoma cells [22, 25, 26, 38, 39] but promotes oncogenic activities in carcinomas where this ratio is low such as in the triple-negative MDA-MB-231, breast cancer cells which lack estrogen receptors, progesterone receptors and the receptor tyrosine-protein kinase erbB-2 (ERBB2) [17, 40, 41].

It was recently shown that, in preadipocytes, the classical RA receptor RARγ directly regulates the transcription of KLF2 and that, in turn, KLF2 induces the expression of both RARγ and its cognate lipid-binding protein CRABP2 [14]. KLF2 and the CRABP2/RAR path thus cooperate in mediating the ability of RA to inhibit differentiation of preadipocytes to mature adipose cells. It was also reported that KLF2 suppresses the expression of FABP5 in MCF-7 mammary carcinoma cells [13]. These observations raise the intriguing possibility that the tumor suppressive activities of KLF2 may originate from the ability of this factor to control the transcriptional activity of RA and that it does so by shifting RA signaling from the pro-oncogenic FABP5/PPARβ/δ to the anti-carcinogenic CRABP2/RAR path.

## RESULTS

### KLF2 is downregulated in breast cancer, positively correlates with patient survival, and suppresses carcinoma growth in a xenograft model of breast cancer

**Figure 1:**
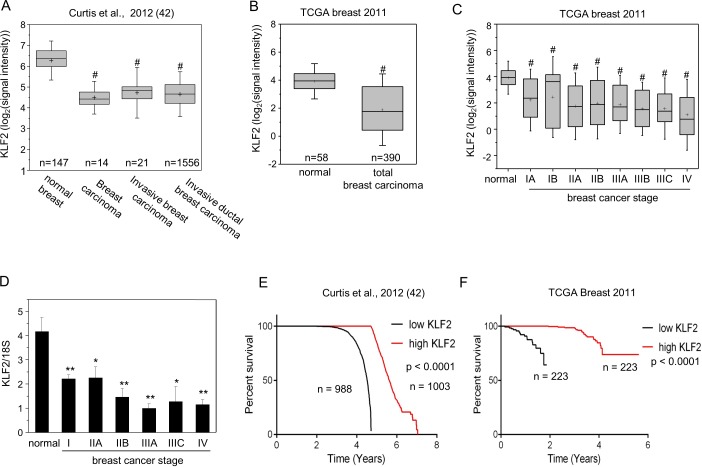
KLF2 is downregulated in human breast tumors and correlated with patients survival **A.** Levels of KLF2 in samples from normal breast, breast carcinoma, invasive breast carcinoma and invasive ductal breast carcinoma reported in [42]. **B.**, **C.** Levels of KLF2 in normal breast tissue and all breast tumors **B.** or tumors at different stages of breast cancer **C.** deposited in The Cancer Genome Atlas (https://tcga-data.nci.nih.gov/tcga/). Group sizes for normal, IA, IB, IIA, IIB, IIIA, IIIB, IIIC and IV were 58, 42, 7, 151, 92, 54, 15, 17, and 12, respectively. Data in **A.**-**C.** were obtained from OncomineTM (Compedia Bioscience, Ann Arbor, Michigan). Whisker indicates S.D., + indicates mean. **p < 0.01, ^#^*p* < 0.0006. **D.** Levels of KLF2 in TissueScanTM tissue qPCR array consisting of cDNA derived from samples of normal breast and denoted stages of breast tumors (OriGene). Mean±SD. *p < 0.05, **p < 0.01 *vs.* normal tissue. **E.**, **F.** Expression levels of KLF2 and their correlations with the survival rate of patients with breast cancer in two studies. Data were obtained from OncomineTM.

Two independent expression array profiles deposited in Oncomine^TM^ Compedia Bioscience [42, 43] documented that the level of KLF2 mRNA is markedly lower in human breast tumors *vs.* normal breast tissue (Figure 1A, 1B). The data further show that KLF2 is downregulated in early stage and remains low at all stages of breast cancer (Figure 1C). Our analysis of a TissueScan^TM^ human breast cancer cDNA array (OriGene) similarly showed marked downregulation of KLF2 at early stage of breast cancer (Figure 1D). Notably, the deposited data [42, 43] show that high mRNA level of KLF2 correlates with markedly better survival rates of breast cancer patients (Figure 1E, 1F).

**Figure 2:**
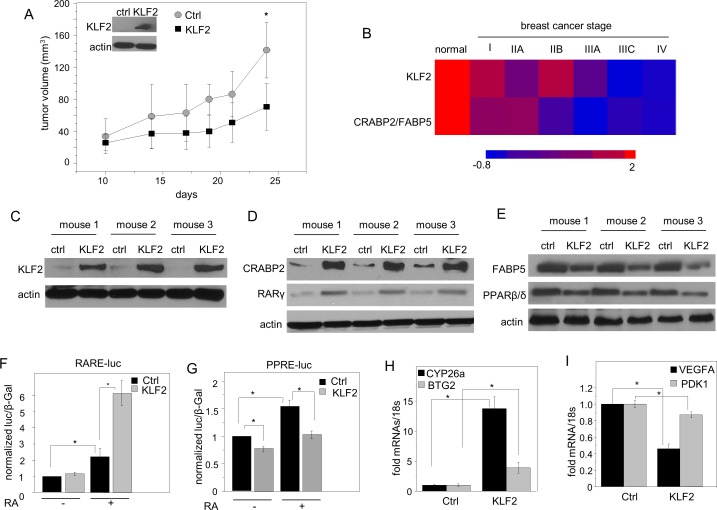
KLF2 is involved in regulating RA signaling **A.** NCr athymic female mice were injected with 5×10^6^ MDA-MB-231 cells into the right flank and cells stably expressing KLF2 into the left flank. Tumor growth at both injection sites was monitored by measuring the length and width with calipers and tumor volume calculated as (length x width^2^)/2. Data are mean±S.D. (*n* = 10) *p < 0.05 vs. control (ctrl) tumors by Paired Student's T-test. Inset: Immunoblots demonstrating stable over-expression of KLF2. **B.** Changes in expression of KLF2 and in the CRABP2/FABP5 ratio during breast cancer progression. Data were obtained by analyzing TissueScanTM tissue qPCR array consisting of cDNA derived from samples of normal breast and denoted stages of breast tumors (OriGene). Expression of KLF2, CRABP2 and FABP5 mRNA was normalized to 18s. **C.** Immunoblots of KLF2 in tumors that arose from parental and KLF2-overexpressing cells. **D.** Immunoblots of CRABP2 and RARγ in tumors that arose from parental and KLF2-overexpressing cells. **E.** Immunoblots of FABP5 and PPARβ/δ in tumors that arose from parental and KLF2-overexpressing cells. **F.**, **G.** MDA-MB-231 cells stably overexpressing GFP (Ctrl) or GFP-KLF2 (KLF2) were co-transfected with vector harboring a luciferase reporter driven by RAR response element (RARE-Luc) **F.** or PPAR response element (PPAR-Luc) **G.** and a vector encoding β-galactosidase, serving as a transfection control. Transactivation assays were carried out in the absence and presence of RA (50 nM for RARE; 200 nM for PPRE). Luciferase activity was normalized to β-galactosidase. Mean±S.D., *n* = 3. *p < 0.05, paired Student's T-test. **H.** Levels of mRNA for BTG2 and CYP26a in tumors that arose from parental and KLF2-overexpressing cells were measured by Q-PCR. Mean±SD, *n* = 3. *p < 0.05, paired Student's T-test. **I.** Levels of mRNA for VEGFA and PDK1 in tumors that arose from parental and KLF2-overexpressing cells were measured by Q-PCR. Mean±SD, *n* = 3. *p < 0.05, paired Student's T-test.

A xenograft mouse model of breast cancer was used to further examine the involvement of KLF2 in mammary tumor development. MDA-MB-231 triple negative mammary carcinoma cells, and an MDA-MB-231 cell line that stably over-expresses KLF2 (Figure 2A, inset) were injected into NCr athymic mice and tumor growth was monitored. To minimize variability between animals, each mouse was injected with the parental cells into one flank, and KLF2-overexpressing cells into the opposite flank. The rate of development of tumors that arose in sites injected with cells that over-express KLF2 was significantly slower as compared with sites injected with parental cells (Figure 2A). Remarkably, all sites injected with parental cells developed tumors, but 2 out of 10 mice injected with KLF2-overexpressing cells remained tumor-free throughout the experiment.

### KLF2 regulates RA signaling

Analysis of a qPCR array consisting of cDNA derived from samples of normal breast and various stages of human breast tumors (OriGene) showed that downregulation of KLF2 during disease progression is correlated with a marked decrease in the ratio of CRABP2 and FABP5 mRNAs (Figure 2B). Measurements of expression levels of RA binding proteins and receptors in tumors that arose in the athymic mice showed that tumors that overexpressed KLF2 (Figure 2C) displayed higher levels of CRABP2 and the RAR isotype RARγ (Figure 2D, Figure S1A) and lower levels of FABP5 and PPARβ/δ (Figure 2E, Figure S1A) as compared with tumors that arose from parental MDA-MB-231 cells. Similarly to effects observed in the tumors, ectopic expression of KLF2 increased the expression of CRABP2 and RARγ and decreased the levels of FABP5 and PPARβ/δ in MDA-MB-231 cells grown in culture (Figure S1B-S1E). The observations thus suggest that KLF2 triggers a switch in RA signaling from the growth-inhibitory CRABP2/RAR pathway to the pro-oncogenic FABP5/PPARβ/δ path. Transcriptional activation assays were carried out to directly examine effects of KLF2 on the transcriptional activity of RAR and PPARβ/δ. MDA-MB-231 cells stably express GFP-tagged KLF2 or GFP alone (Figure S1B) were depleted of retinoids by culturing in charcoal-treated medium. Cells were co-transfected with a vector encoding an RARE-driven or A PPARE-driven luciferase reporter and a vector encoding β-galactosidase, serving as a transfection control. Considering that RA associates with CRABP2 and RAR with a higher affinity than with FABP5 and PPARβ/δ [23, 44, 45], the hormone was used at 50 nM and 200 nM in RARE-driven and in PPARE-driven transactivation assay, respectively. RA-induced activation of the RARE-driven luciferase was significantly enhanced upon ectopic overexpression of KLF2 (Figure 2F). In contrast, KLF2 inhibited RA-induced activation of a PPRE-driven luciferase reporter (Figure 2G). In accordance with activation of RAR and suppression of PPAR-mediated activity by KLF2, analyses of cultured cells (Figure S1F-S1I) and of tumors that arose in NCr mice showed that overexpression of KLF2 upregulated levels of mRNA of the endogenous the RAR target genes *BTG2* [26] and *CYP26a* [46, 47] (Figure 2H). In contrast, levels of mRNAs of two direct PPARβ/δ target genes, the growth factor *VEGFA* [37] and the survival factor *PDK1* [48], were lower in KLF2-expressing tumors (Figure 2I).

**Figure 3:**
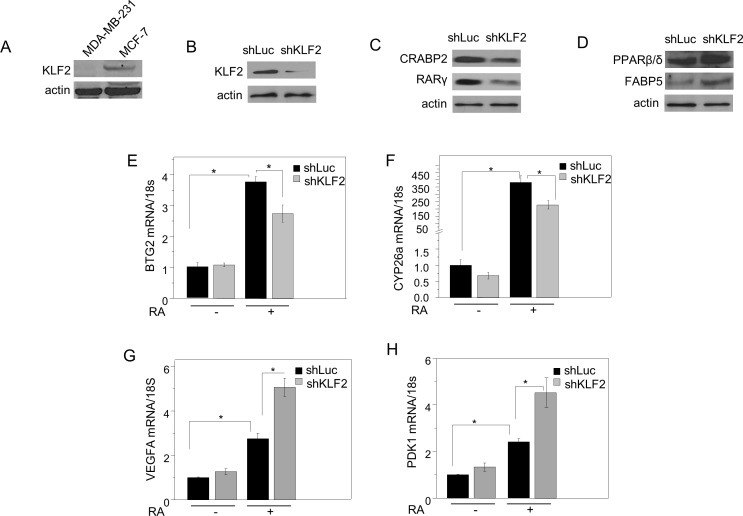
KLF2 regulates RA signaling in MCF-7 mammary carcinoma cells **A.** Expression of KLF2 in MDA-MB-231 and MCF-7 cells examined by immunoblots. **B.** Immunoblots demonstrating down-regulation of KLF2 in MCF-7 cells transfected with vectors harboring shRNA luciferase (shLuc) or shRNA KLF2 (shKLF2). **C.** immunoblots demonstrating expression of CRABP2 and RARγ in MCF-7 cells expressing shLuc or shKLF2. **D.** immunoblots demonstrating expression of FABP5 and PPARβ/δ in MCF-7 cells expressing shLuc or shKLF2. **E.**, **F.** MCF-7 cells expressing shLuc or shKLF2 were treated with vehicle or RA (1 μM) for 4 h. Levels of mRNA for BTG2 **E.** and CYP26a **F.** were measured by Q-PCR. Mean±SD, *n* = 3. *p < 0.05, paired Student's T-test. **G.**, H) MCF-7 cells expressing shLuc or shKLF2 were treated with vehicle or RA (1 μM) for 4 h. Levels of mRNA for VEGFA **G.** and PDK1 **H.** were measured by Q-PCR. Mean±SD, *n* = 3. *p < 0.05, paired Student's T-test.

Unlike MDA-MB-231 cells, MCF-7 mammary carcinoma cells express a high level of KLF2 (Figure 3A). Downregulating KLF2 levels in these cells (Figure 3B) decreased the expression of CRABP2 and RARγ (Figure 3C, Figure S2A) and upregulated FABP5 and PPARβ/δ (Figure 3D, Figure S2B). Accordingly, down-regulation of KLF2 attenuated the ability of RA to induce the expression of the RAR target genes *BTG2* and *Cyp26a* (Figure 3E, 3F), increased the expression of the PPARβ/δ targets *VEGFA* and *PDK1*, and potentiated the ability of RA to induce the expression of these genes (Figure 3G, 3H).

### KLF2 directly controls transcription of CRABP2 and RARγ and indirectly suppresses FABP5

**Figure 4:**
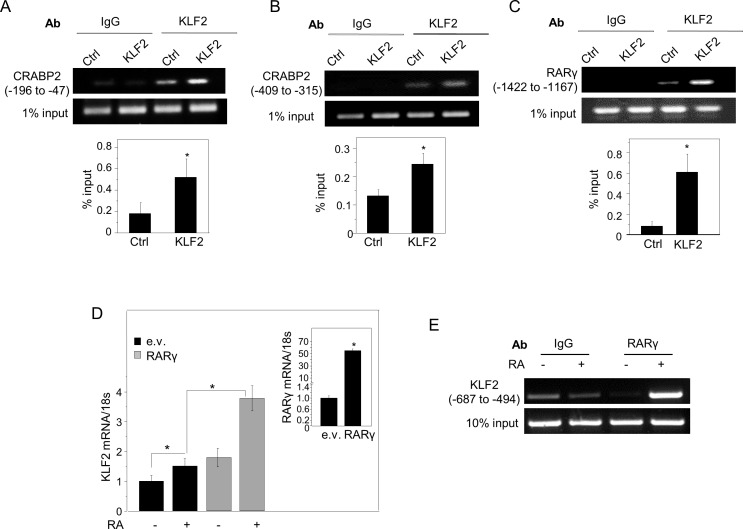
KLF2 directly induces the expression of CRABP2 and RARγ, and RARγ directly regulates KLF2 transcription **A.**-**C.** ChIP assays were carried out using MDA-MB-231 cells stably over-expressing GFP or KLF2. Immunoprecipitated DNA was amplified using primers specific for the putative KLF2 response elements of the CRABP2 promoter **A.**, **B.** or the putative KLF2 element in the promoter of RARγ **C. D.** MDA-MB-231, transiently expressing a control vector or a vector encoding RARγ were treated with RA (1 μM) or vehicle for 4 h. KLF2 mRNA levels were measured by Q-PCR. Mean±S.D. (*n* = 3). *p < 0.05, paired Student's T-test. Inset: levels of RARγ mRNA in cells transfected with an empty vector or a vector encoding RARγ, assessed by Q-PCR. **E.** ChIP assays demonstrating RA-induced recruitment of RARγ to the RARE-containing region of the KLF2 promoter.

Examination of the promoter region of CRABP2 revealed CACCC motifs, corresponding to consensus KLF2 response elements [11], at 74, 105, 165, 365, 373, 666, 784, 1062, and 1172 basepairs (bp) upstream from the transcription start site. Chromatin immunoprecipitation (ChiP) assays demonstrated that endogenous KLF2 is recruited to the region that encompasses the first three elements (Figure 4A) as well as to a region that includes the REs at −365 and −373 bp RE (Figure 4B). Ectopic over-expression of KLF2 enhanced the response (Figure 4A, 4B). Examination of the promoter of RARγ similarly revealed several putative KLF2 REs. ChiP assays showed that, of these, endogenous KLF2 was associated with a region that contains RE at 1192 and 1397 bp upstream from the start site and ectopic overexpression of the factor enhanced the response (Figure 4C). The observations indicate that KLF2 regulates the expression of CRABP2 and RARγ by directly binding to their promoters in MDA-MB-231 cells,.

Interestingly, treatment with RA induced the expression of KLF2, and ectopic overexpression of RARγ enhanced the response (Figure 4D). ChiP assays showed that RA triggered recruitment of RARγ to a direct repeat 2 (DR-2) RARE present at 687 bp upstream from the KLF2 transcription start site (Figure 4E), indicating that RARγ directly controls KLF2 transcription. Hence, KLF2 and RARγ appear to form a positive feedback loop through which they promote each other's expression.

**Figure 5:**
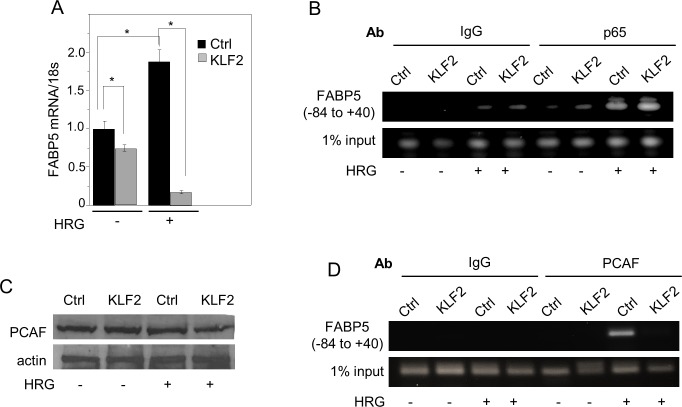
KLF2 suppresses FABP5 by interfering with the transcriptional activity of NFκB at the FABP5 promoter **A.**. MDA-MB-231 cells stably over-expressing GFP or KLF2 were serum starved overnight, treated with vehicle or heregulin-β1 (HRG, 30 μg/ml) for 4 h. and level of FABP5 mRNA was measured by Q-PCR. Mean±SD (*n* = 3). *p < 0.05. **B.**, **D.** ChIP assays were carried out using MDA-MB-231 cells stably over-expressing GFP or KLF2 untreated or treated with HRG with presence or absence of HRG (30 μg/mL, 24 h). Immunoprecipiations was carried out using non-specific IgG or antibodies against the NFκB subunit p65 **B.** or antibodies against PCAF **D.** Immunoprecipitated DNA was amplified using primers for the NFκB response element of the FABP5 promoter. **C.** Immunoblots demonstrating expression level of PCAF in MDA-MB-231 cells stably over-expressing GFP or KLF2 in the absence or presence of HRG (30 μg/ml, 24 h).

Several putative KLF2 REs appear to be present in the promoter of FABP5 but ChiP assays indicated that these are not functional in recruiting KLF2 in MDA-MB-231 cells (data not shown). KLF2 may thus suppress FABP5 expression by an indirect mechanism. It was previously reported that KLF2 inhibits the transcriptional activity of NF-κB by sequestering the transcriptional coactivator p300/CBP-associated factor (PCAF) [49]. Considering the report that FABP5 expression is regulated by NF-κB [13], we wondered whether such a mechanism underlies the observed suppression of FABP5 expression by KLF2. To activate NF-κB, cells were treated with the growth factor heregulin-β1 (HRG). Similarly to the response previously observed in MCF-7 cells [13], HRG induced the expression of FABP5 in MDA-MB-231 cells (Figure 5A). Ectopic expression of KLF2 lowered the level of FABP5 mRNA both in the absence and in the presence of HRG (Figure 5A). Activation by HRG resulted in recruitment of the NF-κB p65 subunit to the NF-κB RE located 49 bp upstream from the transcription start site in the FABP5 promoter (Figure 5B). Neither HRG treatment nor ectopic expression of KLF2 altered the total expression level of PCAF (Figure 5C). However, while PCAF effectively associated with the NF-κB RE at the FABP5 promoter upon its activation by HRG, no such recruitment was observed in the presence of KLF2 (Figure 5D). Hence, KLF2 suppresses FABP5 expression by interfering with the recruitment of PCAF to NF-kB at the FABP5 promoter.

### KLF2 converts RA from a pro-oncogenic to an anti-oncogenic agent

**Figure 6:**
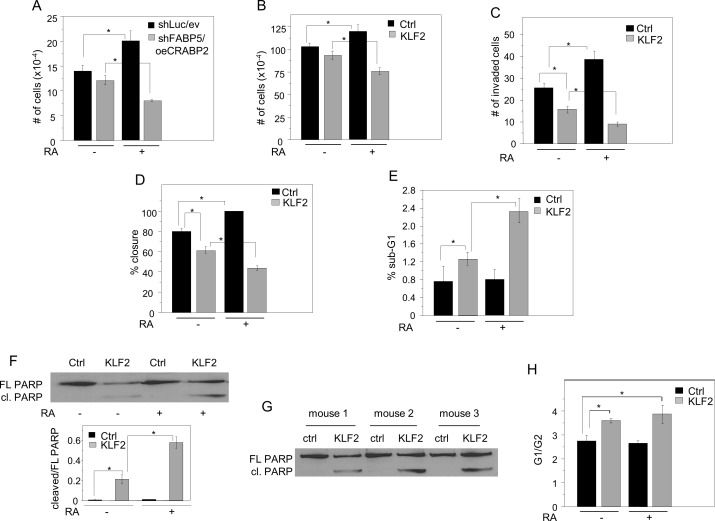
KLF2 converts RA from a pro-oncogenic to an anti-oncogenic agent **A.** MDA-MB-231 cells were transfected with control vectors encoding shLuc and empty vector (ev), or with vectors harboring shFABP5 and CRABP2. Cells were cultured in medium containing 10% charcoal-treated FBS, treated with vehicle or RA (1 μM) for 4 days, and counted. *p < 0.05 (*n* = 3) by paired Student's T-test. **B.** MDA-MB-231 cells stably overexpressing GFP (Ctrl) or GFP-KLF2 (KLF2) were cultured in medium containing 10% charcoal-treated FBS, treated with vehicle or RA (1 μM) for 4 days, and counted. *p < 0.05 (*n* = 3) by paired Student's T-test. **C.** Invasion assays using cells stably overexpressing GFP (Ctrl) or KLF2 and treated with RA (1 μM, 24 h.) or vehicle. Mean±S.D. (*n* = 3). *p < 0.05, paired Student's T-test. **D.** Scratch assays using cells stably overexpressing GFP (Ctrl) or KLF2 and treated with RA (1 μM) or vehicle. Quantitation of % closure after 24 h is shown. Mean±S.D. (*n* = 3). *p < 0.05, paired Student's T-test. (See Figure S4 for representative images). **E.** MDA-MB-231 cells that stably overexpress GFP (Ctrl) or KLF2 were cultured in medium containing 10% charcoal-treated FBS in the presence of vehicle (ethanol) or RA (1 μM) for 5 days. RA was replenished every 24 h. Cells were stained with propidium iodide and fractions of cells in different cell cycle stages assessed by FACS. The fraction of cells with fragmented DNA (sub-G1) is shown. Mean±S.D. (*n* = 3). *p < 0.05, paired Student's T-test. **F.** Top: MDA-MB231 cells stably overexpressing GFP (Ctrl) or KLF2 were cultured in medium containing 10% charcoal-treated FBS in the absence or presence of RA (1 μM) for 5 days. RA was replenished every 24 h. PARP and cleaved PARP were assessed by immunoblots. Bottom: quantitation of the cleaved-PARP/total-PARP ratio. Mean±S.D. (*n* = 3). *p < 0.05, paired Student's T-test *p < 0.05. **G.** Immunoblots demonstrating levels of PARP and cleaved PARP in tumors that arose from parental and KLF2-overexpressing MDA-MB-231 cells. **H.** MDA-MB-231 cells that stably overexpress GFP (Ctrl) or KLF2 were cultured in medium containing 10% charcoal-treated FBS in the presence of vehicle (ethanol) or RA (1 μM) for 5 days. RA was replenished every 24 h. Cell cycle stages were assessed by FACS. Mean±S.D. (*n* = 3). *p < 0.05, paired Student's T-test.

MDA-MB-231 cells express a high FABP5/CRABP2 ratio and, consequently, RA promotes their growth (Figure 6A). To decrease this ratio, FABP5 expression was downregulated using two different shRNAs and, concomitantly, CRABP2 was ectopically overexpressed (Figure S3A). In contrast with its effect on the growth of parental MDA-MB-231 cells, RA suppressed the growth of cells which express a low FABP5/CRABP2 ratio (Figure 6A, Figure S3B). In accordance with the notion that the tumor suppressing activity of KLF2 is exerted at least in part by controlling RA signaling, ectopic expression of this factor, similarly to direct alteration of the FABP/CRABP2 ratio, converted RA from a pro- to an anti-proliferative agent (Figure 6B, Figure S3C). In addition to promoting cell proliferation, RA enhanced cell invasion through a matrix gel (Figure 6C) and facilitated wound closure in scratch assays (Figure 6D, Figure S3D). Conversely, in KLF2-overexpressing cells, RA suppressed cell invasion and inhibited wound closure (Figure 6C, 6D, Figure S3D). KLF2 displayed a modest apoptotic activity on its own but it markedly potentiated RA-induced apoptosis (Figure 6E, 6F, Figure S3D). Similarly to the response of cultured cells, KLF2-overexpressing tumors that arose in NCr mice (Figure 2A) displayed a higher apoptotic status, reflected by PARP cleavage (Figure 6G). FACS analysis (Figure 6H, Figure S4) showed that, in parental cells, 57.6±11.6% and 59.9.8±10.1% were in G1 phase and 20.8±2.2% and 22.5±3.3% were in G2 phase in the absence and presence of RA, respectively. Interestingly, overexpression of KLF2 shifted the distribution resulting in 63.4±9.4% and 17.6±2.9% of cells placed in G1 and G2 phases, respectively. Similarly to parental cells, this distribution was not affected by RA treatment (Figure 6H). These observations thus show that KLF2 expression resulted in a G1 arrest even in the absence of RA and suggest that, in addition to regulating RA signaling, KLF2 also exerts additional RA-independent antiproliferative activities.

## DISCUSSION

**Figure 7:**
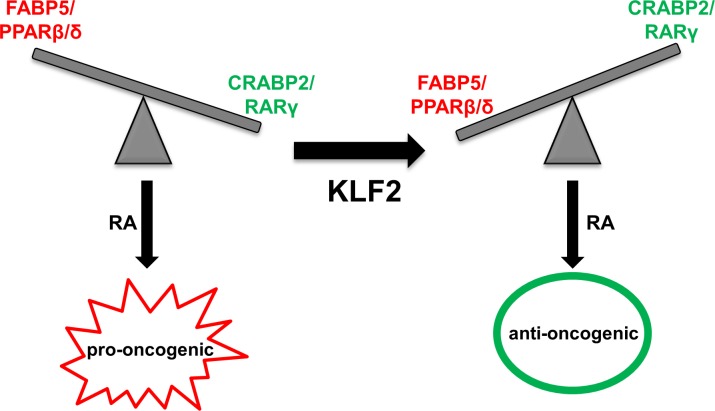
A model outlining cross-talk between KLF2 and RA signaling in control of cancer cell growth In cancer cells that express a high FABP5/CRABP2 ratio, RA is ‘channeled’ to PPARβ/δ and promotes tumorigenesis. KLF2 expression results in decreased expression of FABP5 and PPARβ/δ and in upregulation of CRABP2 and RAR. RA is thus diverted from a pro-oncogenic to an anti-oncogenic pathway and suppresses tumorigenesis.

The observations reveal that KLF2 controls the transcriptional activity of RA and that it does so by regulating the expression of RA nuclear receptors RAR and PPARβ/δ and their cognate lipid-binding proteins CRABP2 and FABP5 (Figure 7). KLF2 directly binds to response elements in the proximal promoters of CRABP2 and RARγ and upregulates their expression. Concomitantly, KLF2 suppresses the expression of FABP5 and PPARβ/δ. In accordance with the previous report that KLF2 inhibits the transcriptional activity of NF-κB by “squelching” the transcriptional coactivator PCAF [49], the data indicate that KLF2 suppresses the expression of FABP5 by interfering with recruitment of PCAF to NF-κB at the FABP5 promoter. The mechanism by which KLF2 inhibits the expression of PPARβ/δ remains to be clarified. The data further show that, in MDA-MB-231 cells, KLF2 is directly regulated by RARγ. Hence, KLF2 and RARγ form a positive feedback loop through which they promote each other's expression.

By upregulating CRABP2 and RARγ and decreasing the expression of FABP5 and PPARβ/δ, KLF2 diverts RA signaling from a pro-oncogenic to an anti-proliferative path, and the positive feedback loop between KLF2 and RARγ further amplifies this activity. Indeed, while RA promoted proliferation, migration, and invasion in parental MDA-MB-231, expression of KLF2 converted the hormone to an agent that suppressed growth and oncogenic properties and triggered apoptosis in cultured cells and in tumors that arose in a xenograft mouse model of breast cancer. Hence, by controlling the partitioning of RA between its two transcriptional pathways, KLF2 potently overcame the profound RA-resistance of highly metastatic, triple negative breast cancer cells. It is worth noting that KLF2 inhibited cancer cell invasion and migration, and induced apoptosis and cell cycle arrest even with the absence of RA. These observations indicate that, besides potentiating the anticarcinogenic activities of RA, KLF2 suppresses tumor growth by additional, RA-independent, mechanism(s) the nature of which remains to be clarified.

The molecular events that lead to suppression of KLF2 during breast cancer development are incompletely understood but it is worth noting that it has been reported that KLF2 expression is inhibited by NF-κB [13]. Considering that NF-κB is activated by epidermal growth factor receptors (EGFR) [13], the amplification of the EGFR gene *HER2/ERBB2/neu* in a significant fraction of human breast cancers [50] may provide a partial explanation for downregulation of KLF2 in breast tumors. Taken together, the data show that KLF2 appreciably contributes to the development of some human breast cancers and point at this factor and its downstream effectors, RA binding proteins and nuclear receptors, as novel targets for therapy of some human breast cancers.

## MATERIALS AND METHODS

### Reagents

RA was purchased from Calbiochem (Millipore Corp). Antibodies against FABP5 (AF1476) and PPARβ/δ (AB10094) were obtained from R&D Systems and Millipore Corp., respectively. Antibodies against RARγ (sc-7387), actin (sc-47778), PCAF (sc-8999), p65 (sc-372), and KLF2 (sc-28675) were purchased from Santa Cruz. Antibodies against PARP (9532) were from Cell Signaling. Anti-mouse (170-6516) and anti-rabbit (170-6515) immunoglobulin horseradish peroxidase-conjugated antibodies were from BioRad. Antibodies against CRABP2 were kindly provided by Cecile Rochette-Egly (IGBMC, Strasbourg, France). Normal mouse (sc-2025) and rabbit (sc-2027) IgG for the Chip control were purchased from Santa Cruz.

### Cells

MDA-MB-231 cells were maintained in Dulbecco's Modified Eagle's Medium (DMEM) supplemented with 10% fetal bovine serum (FBS) and penicillin/streptomycin (100 units/ml). To generate cells stably over-expressing KLF2, MDA-MB-231 cells were infected with lentivirus encoding GFP (ctrl) or GFP-KLF2 (KLF2) (Welgen Inc.) and selected using puromycin (0.7 μg/ml). Individual colonies were pooled. To flip CRABP2/FABP5 ratio in cells, MDA-MB-231 cells were transiently infected with a vector harboring shRNA FABP5 (shFABP5 1: TRCN0000059698; shFABP5 2: TRCN0000059700, GE Dharmacon) together with an expression vector encoding CRABP2 (A vector encoding CRABP2 was generated by inserting cDNA for human CRABP2 into BamHI and EcoRI sites of pCMV-3Tag-1 vector). To generate KLF2 knock-down cells, MCF-7 cells were transiently infected with lentivirus encoding shRNA luciferase (control) or shRNA KLF2 (shKLF2 1: TRCN0000020725; shKLF2 2: TRCN0000020726, GE Dharmacon).

### Immunoblotting

Cells were lysed in a RIPA buffer containing 150 mM NaCl, 10 mM Tris, pH 7.2, 0.1% SDS, 1% Triton X-100, 1% deoxycholate, 5 mM EDTA, 1 mM PMSF, 2 μg/ml leupeptin, 2 μg/ml aprotinin and 2 μg/ml pepstatin A. Protein concentration was determined by the Bradford protein assay. Cell lysates (50 μg protein) were resolved by SDS-PAGE and immunobloted using appropriate antibodies.

### Quantitative real-time PCR (Q-PCR)

Total RNA was extracted using Trizol. 2 μg mRNA was reverse transcribed into cDNA using the high capacity RNA to cDNA kit from Applied Biosystems (Gaithersburg, MD). Quantitative real-time PCR (Q-PCR) analyses were performed in tripicates using the Taqman Gene Expression Master Mix (Applied Biosystems) and TaqMan chemistry and Assays on Demand probes: FABP5 (Hs00154260-m1), CRABPII (Hs00154260-m1), RARγ (Hs01559234-m1), PPARβ/δ (Hs00606407_m1), RARβ (Hs00977140-m1), VEGFA (Hs00173626-m1), CYP26A1 (Hs 00175627-m1), PDK1 (Hs00765634-m1), BTG2 (Hs00198887_m1) and KLF2 (Hs00360439-g1). As internal control, 18s rRNA (4319413E-0710034) was used.

### Chromatin immunoprecipitation (ChIP) assays

Cells were grown to 70-80% confluency on 5×100 mm^3^ tissue culture dishes. Cells were fixed with 1% formaldehyde (15 min.), quenched by the addition of 0.125 M glycine (5. min.), washed three times with PBS, scraped in PBS and centrifuged. Cells were resuspended in buffer containing 5 mM PIPES, pH 8.0, 85 mM KCl, 0.5% NP40, 10 μg/ml leupeptin, 1 μg/ml aprotinin, and 5 μg/ml pepstatin A, pelleted, resuspended in a buffer containing 10 mM Tris-HCl pH 8.1, 10 mM EDTA, 1% SDS, 1 μg/ml leupeptin, 1 μg/ml aprotinin, and 5 μg/ml pepstatin A. Cells were sonicated to yield DNA fragments 250-1000 bp in size. Sonicated samples were diluted 10 fold in dilution buffer (0.01% SDS, 1.1% Triton X 100, 1.2 mM EDTA, 16.7 mM Tris-HCl pH 8.1, 167 mM NaCl) and incubated for 1 h at 4°C with 50 μl/ml of 50% slurry of Protein A-Sepharose. Supernatant was transferred to a tube containing 10 μg antibodies. See Experimental Procedures for antibodies used. After overnight incubation, beads were centrifuged and washed sequentially with 1 ml of buffer containing 0.1% SDS and 150 mM NaCl, 1 ml of buffer containing 0.1% SDS and 500 mM NaCl, 1 ml of buffer containing 10 mM Tris, pH 8.1, 0.25 M LiCl, 1% Nonidet P-40, 1% sodium deoxycholate, and 1 mM EDTA, and two washes with 1 ml of buffer containing 10 mM Tris, pH 8.0, and 1 mM EDTA. 200 μl of elution buffer (0.1 M NaHCO_3_ and 1% SDS) were added to the beads, and samples were incubated at room temperature for 15 min with agitation. Eluates were reverse crosslinked overnight at 65°C. The supernatant was incubated for 1 hour at 45°C with 1 M Tris 6.5 and 10 mg/ml proteinase K. DNA was extracted using phenol-chlorofrom. PCRs were carried out using Go Taq DNA polymerase (Promega). The regions that contained KLF2 response elements regions (CACCC) were amplified by PCR using the following primers: CRABP2 forward 5′-AGC TAC GGC TCA AGA TCT GG-3′ and reverse 5′-GGG CTC GTG TAT GGC TG-3′; CRABP2 forward 5′-TTC CAG AGT CCC CAG GCA-3′ and reverse 5′-GCT GGA ACA ACT CGG AGA GG-3′; RARγ forward 5′-TGG AGT GAA AGA GAG GGC-3′ and reverse 5′-CTT CCC CAG CAA TGC TCGA-3′. The regions that contained p65 response elements regions were amplified by PCR using the following primers: FABP5 forward 5′-CAC CTC CCG ACC CCG AGAA-3′ and reverse 5′-CCG GCG GCT GCT TTA TAA CG-3′. Primers used for detecting RARγ on KLF2 promoter: forward 5′-CCC ACC TCA GCC TCC CAC TAC ACC CAGC-3′ and reverse 5′-GAT GGA TGG GAA GTC TGG AGT CTC CAG GAT TCA TGG-3′. PCR products was separated on a 1% agarose gel, stained in ethidium bromide, and visualized with an AlphaImager® HP System.

### Fluorescence activated cell sorting (FACS)

Cells were seeded in 60 mm plates in DMEM supplemented with 10% charcoal-treated FBS. RA (1 μM) was replenished every 24 hr. for 5 days. Cells were collected, washed with PBS and fixed in 70% ice-cold ethanol overnight. Cells were stained with propidium iodide (0.5 μg/ml) containing 100 U/ml RNASe for 30 min. Samples were analyzed at the Case Western Reserve University cell sorting facility, using a Becton Dickinson LSRII cell sorter.

### Cell proliferation assays

3 x10^4^ cells were plated in triplicates in a 6-well plate and treated with RA (1μM) for 4 days. Ligand was replaced daily. Cell growth was assessed by counting.

### Wound healing assays

70 μL of 5 × 10^5^ cells/ml cells were seeded into 35mm μ-Dish culture-insert (80206, Ibidi, Germany). Cells were grown to the confluent layer overnight.. The insert was gently removed without touching attached cells, and cells were washed extensively with PBS 3 times to remove floating cells, and medium with vehicle (EtOH) or RA (1 μM) was replaced into culture dish. Images were taken at initial and 24 h after insert removed..

### Invasion assays

Invasion assays were performed using BD bioscience invasion assay following manufacture's protocol. Briefly, 5×10^4^ cells/mL were placed in a 24 well plate containing matrigel and grown for 24 h. Medium was aspirated and excess cells removed using a cotton swab. Membranes were then stained using the Diff-Quik staining kit (IMBC Inc), excised, mounted on a microscope slide and cells were counted.

### Transactivation assays

2×10^5^ cells were plated in 6-well plates in DMEM supplemented with 10% charcoal-treated FBS. Cells were transfected with vectors harboring a luciferase reporter driven by a PPAR RE (PPRE-Luc) or an RAR RE (RARE-Luc). Cells were co-transfected with an expression vector for β-galactosidase, serving as transfection efficiency control. 24 h post-transfection, cells were treated with 0.2 μM RA (50 nM RA for RAR) or vehicle overnight. Cells were lysed, and luciferase activity was assayed using the luciferase assay buffer (Promega) and corrected for transfection efficiency by the activity of β-galactosidase.

### Mice studies

NCr athymic mice were housed in accordance with ARC protocol and IACUC regulations. Eight-week old NCr athymic female mice (Athymic Animal & Xenograft Core Facility, Case Western Reserve University) were injected with 5×10^6^ MDA-MB-231 cells or cells that stably overexpress KLF2. Parental and KLF2-overexpressing cells in PBS were injected into left and right mammary fat pad of the same animal, respectively. Tumor development was monitored by measuring the length and width with calipers and tumor volume calculated as (length x width)^2^/2. Tumors were collected at termination for mRNA and protein expression evaluation.

### Human breast cancer cDNA array

cDNA array was purchased from OriGene (BCRT101). Samples were analyzed by Q-PCR. Taqman probes for KLF2, CRABP2 and FABP5 were from Applied Biosystems (Hs00360439, Hs00275636, and Hs02339439, respectively).

### Statistical analysis

Statistical significance of difference in KLF2 expression between breast tumors and normal tissue was analyzed by an unpaired T-test. Statistical analyses on xenograft tumors were carried out by Paired Student's T-test. Analyses were performed using SPSS 16.0 software

## SUPPLEMENTARY MATERIAL FIGURES



## References

[1] Black AR, Black JD, Azizkhan-Clifford J (2001). Sp1 and kruppel-like factor family of transcription factors in cell growth regulation and cancer. Journal of cellular physiology.

[2] Kaczynski J, Cook T, Urrutia R (2003). Sp1- and Kruppel-like transcription factors. Genome biology.

[3] Liu R, Zheng HQ, Zhou Z, Dong JT, Chen C (2009). KLF5 promotes breast cell survival partially through fibroblast growth factor-binding protein 1-pERK-mediated dual specificity MKP-1 protein phosphorylation and stabilization. The Journal of biological chemistry.

[4] Frigo DE, Sherk AB, Wittmann BM, Norris JD, Wang Q, Joseph JD, Toner AP, Brown M, McDonnell DP (2009). Induction of Kruppel-like factor 5 expression by androgens results in increased CXCR4-dependent migration of prostate cancer cells in vitro. Molecular endocrinology.

[5] Dang DT, Chen X, Feng J, Torbenson M, Dang LH, Yang VW (2003). Overexpression of Kruppel-like factor 4 in the human colon cancer cell line RKO leads to reduced tumorigenecity. Oncogene.

[6] Fernandez-Zapico ME, Lomberk GA, Tsuji S, DeMars CJ, Bardsley MR, Lin YH, Almada LL, Han JJ, Mukhopadhyay D, Ordog T, Buttar NS, Urrutia R (2011). A functional family-wide screening of SP/KLF proteins identifies a subset of suppressors of KRAS-mediated cell growth. The Biochemical journal.

[7] Fernandez-Zapico ME, Mladek A, Ellenrieder V, Folch-Puy E, Miller L, Urrutia R (2003). An mSin3A interaction domain links the transcriptional activity of KLF11 with its role in growth regulation. The EMBO journal.

[8] Kremer-Tal S, Narla G, Chen Y, Hod E, DiFeo A, Yea S, Lee JS, Schwartz M, Thung SN, Fiel IM, Banck M, Zimran E, Thorgeirsson SS, Mazzaferro V, Bruix J, Martignetti JA (2007). Downregulation of KLF6 is an early event in hepatocarcinogenesis, and stimulates proliferation while reducing differentiation. Journal of hepatology.

[9] Duhagon MA, Hurt EM, Sotelo-Silveira JR, Zhang X, Farrar WL (2010). Genomic profiling of tumor initiating prostatospheres. BMC genomics.

[10] Wang F, Zhu Y, Huang Y, McAvoy S, Johnson WB, Cheung TH, Chung TK, Lo KW, Yim SF, Yu MM, Ngan HY, Wong YF, Smith DI (2005). Transcriptional repression of WEE1 by Kruppel-like factor 2 is involved in DNA damage-induced apoptosis. Oncogene.

[11] Wu J, Lingrel JB (2004). KLF2 inhibits Jurkat T leukemia cell growth via upregulation of cyclin-dependent kinase inhibitor p21WAF1/CIP1. Oncogene.

[12] Bhattacharya R, Senbanerjee S, Lin Z, Mir S, Hamik A, Wang P, Mukherjee P, Mukhopadhyay D, Jain MK (2005). Inhibition of vascular permeability factor/vascular endothelial growth factor-mediated angiogenesis by the Kruppel-like factor KLF2. The Journal of biological chemistry.

[13] Kannan-Thulasiraman P, Seachrist DD, Mahabeleshwar GH, Jain MK, Noy N (2010). Fatty acid-binding protein 5 and PPARbeta/delta are critical mediators of epidermal growth factor receptor-induced carcinoma cell growth. The Journal of biological chemistry.

[14] Berry DC, DeSantis D, Soltanian H, Croniger CM, Noy N (2012). Retinoic acid upregulates preadipocyte genes to block adipogenesis and suppress diet-induced obesity. Diabetes.

[15] Chambon P (1996). A decade of molecular biology of retinoic acid receptors. FASEB journal : official publication of the Federation of American Societies for Experimental Biology.

[16] Germain P, Chambon P, Eichele G, Evans RM, Lazar MA, Leid M, De Lera AR, Lotan R, Mangelsdorf DJ, Gronemeyer H (2006). International Union of Pharmacology. LX. Retinoic acid receptors. Pharmacol Rev.

[17] Schug TT, Berry DC, Shaw NS, Travis SN, Noy N (2007). Opposing effects of retinoic acid on cell growth result from alternate activation of two different nuclear receptors. Cell.

[18] Shaw N, Elholm M, Noy N (2003). Retinoic acid is a high affinity selective ligand for the peroxisome proliferator-activated receptor beta/delta. The Journal of biological chemistry.

[19] Tan NS, Shaw NS, Vinckenbosch N, Liu P, Yasmin R, Desvergne B, Wahli W, Noy N (2002). Selective cooperation between fatty acid binding proteins and peroxisome proliferator-activated receptors in regulating transcription. Molecular and cellular biology.

[20] Sessler RJ, Noy N (2005). A ligand-activated nuclear localization signal in cellular retinoic acid binding protein-II. Molecular cell.

[21] Armstrong EH, Goswami D, Griffin PR, Noy N, Ortlund EA (2014). Structural basis for ligand regulation of the Fatty Acid Binding Protein 5, Peroxisome Proliferator Activated Receptor beta/delta (FABP5-PPARbeta/delta) signaling pathway. The Journal of biological chemistry.

[22] Budhu AS, Noy N (2002). Direct channeling of retinoic acid between cellular retinoic acid-binding protein II and retinoic acid receptor sensitizes mammary carcinoma cells to retinoic acid-induced growth arrest. Molecular and cellular biology.

[23] Dong D, Ruuska SE, Levinthal DJ, Noy N (1999). Distinct roles for cellular retinoic acid-binding proteins I and II in regulating signaling by retinoic acid. The Journal of biological chemistry.

[24] Budhu A, Gillilan R, Noy N (2001). Localization of the RAR interaction domain of cellular retinoic acid binding protein-II. J Mol Biol.

[25] Donato LJ, Noy N (2005). Suppression of mammary carcinoma growth by retinoic acid: proapoptotic genes are targets for retinoic acid receptor and cellular retinoic acid-binding protein II signaling. Cancer research.

[26] Donato LJ, Suh JH, Noy N (2007). Suppression of mammary carcinoma cell growth by retinoic acid: the cell cycle control gene Btg2 is a direct target for retinoic acid receptor signaling. Cancer research.

[27] Noy N (2010). Between death and survival: retinoic acid in regulation of apoptosis. Annual review of nutrition.

[28] Afonja O, Juste D, Das S, Matsuhashi S, Samuels HH (2004). Induction of PDCD4 tumor suppressor gene expression by RAR agonists, antiestrogen and HER-2/neu antagonist in breast cancer cells. Evidence for a role in apoptosis. Oncogene.

[29] Afonja O, Raaka BM, Huang A, Das S, Zhao X, Helmer E, Juste D, Samuels HH (2002). RAR agonists stimulate SOX9 gene expression in breast cancer cell lines: evidence for a role in retinoid-mediated growth inhibition. Oncogene.

[30] Zacheis D, Dhar A, Lu S, Madler MM, Klucik J, Brown CW, Liu S, Clement F, Subramanian S, Weerasekare GM, Berlin KD, Gold MA, Houck JR, Fountain KR, Benbrook DM (1999). Heteroarotinoids inhibit head and neck cancer cell lines in vitro and in vivo through both RAR and RXR retinoic acid receptors. Journal of medicinal chemistry.

[31] Soprano DR, Qin P, Soprano KJ (2004). Retinoic Acid Receptors and Cancers. Annual review of nutrition.

[32] Adachi Y, Itoh F, Yamamoto H, Iku S, Matsuno K, Arimura Y, Imai K (2001). Retinoic acids reduce matrilysin (matrix metalloproteinase 7) and inhibit tumor cell invasion in human colon cancer. Tumour biology : the journal of the International Society for Oncodevelopmental Biology and Medicine.

[33] Di-Poi N, Michalik L, Tan NS, Desvergne B, Wahli W (2003). The anti-apoptotic role of PPARbeta contributes to efficient skin wound healing. J Steroid Biochem Mol Biol.

[34] Di-Poi N, Tan NS, Michalik L, Wahli W, Desvergne B (2002). Antiapoptotic role of PPARbeta in keratinocytes via transcriptional control of the Akt1 signaling pathway. Molecular cell.

[35] Aggarwal BB, Sethi G, Ahn KS, Sandur SK, Pandey MK, Kunnumakkara AB, Sung B, Ichikawa H (2006). Targeting signal-transducer-and-activator-of-transcription-3 for prevention and therapy of cancer: modern target but ancient solution. Annals of the New York Academy of Sciences.

[36] Montagner A, Delgado MB, Tallichet-Blanc C, Chan JS, Sng MK, Mottaz H, Degueurce G, Lippi Y, Moret C, Baruchet M, Antsiferova M, Werner S, Hohl D, Saati TA, Farmer PJ, Tan NS (2014). Src is activated by the nuclear receptor peroxisome proliferator-activated receptor beta/delta in ultraviolet radiation-induced skin cancer. EMBO molecular medicine.

[37] Wang D, Wang H, Guo Y, Ning W, Katkuri S, Wahli W, Desvergne B, Dey SK, DuBois RN (2006). Crosstalk between peroxisome proliferator-activated receptor delta and VEGF stimulates cancer progression. Proceedings of the National Academy of Sciences of the United States of America.

[38] Manor D, Shmidt EN, Budhu A, Flesken-Nikitin A, Zgola M, Page R, Nikitin AY, Noy N (2003). Mammary carcinoma suppression by cellular retinoic acid binding protein-II. Cancer research.

[39] Schug TT, Berry DC, Toshkov IA, Cheng L, Nikitin AY, Noy N (2008). Overcoming retinoic acid-resistance of mammary carcinomas by diverting retinoic acid from PPARbeta/delta to RAR. Proceedings of the National Academy of Sciences of the United States of America.

[40] Levi L, Lobo G, Doud MK, von Lintig J, Seachrist D, Tochtrop GP, Noy N (2013). Genetic ablation of the fatty acid-binding protein FABP5 suppresses HER2-induced mammary tumorigenesis. Cancer research.

[41] Morgan E, Kannan-Thulasiraman P, Noy N (2010). Involvement of Fatty Acid Binding Protein 5 and PPARbeta/delta in Prostate Cancer Cell Growth. PPAR Res.

[42] Curtis C, Shah SP, Chin SF, Turashvili G, Rueda OM, Dunning MJ, Speed D, Lynch AG, Samarajiwa S, Yuan Y, Graf S, Ha G, Haffari G, Bashashati A, Russell R, McKinney S (2012). The genomic and transcriptomic architecture of 2,000 breast tumours reveals novel subgroups. Nature.

[43] TCGA (2011). The Cancer Genome Atlas.

[44] Sussman F, de Lera AR (2005). Ligand recognition by RAR and RXR receptors: binding and selectivity. Journal of medicinal chemistry.

[45] Levi L, Lobo G, Doud MK, von Lintig J, Seachrist D, Tochtrop GP, Noy N Genetic ablation of the fatty acid-binding protein FABP5 suppresses HER2-induced mammary tumorigenesis. Cancer research.

[46] Ray WJ, Bain G, Yao M, Gottlieb DI (1997). CYP26, a novel mammalian cytochrome P450, is induced by retinoic acid and defines a new family. The Journal of biological chemistry.

[47] White JA, Ramshaw H, Taimi M, Stangle W, Zhang A, Everingham S, Creighton S, Tam SP, Jones G, Petkovich M (2000). Identification of the human cytochrome P450, P450RAI-2, which is predominantly expressed in the adult cerebellum and is responsible for all-trans-retinoic acid metabolism. Proceedings of the National Academy of Sciences of the United States of America.

[48] Tan NS, Michalik L, Noy N, Yasmin R, Pacot C, Heim M, Fluhmann B, Desvergne B, Wahli W (2001). Critical roles of PPAR beta/delta in keratinocyte response to inflammation. Genes & development.

[49] Das H, Kumar A, Lin Z, Patino WD, Hwang PM, Feinberg MW, Majumder PK, Jain MK (2006). Kruppel-like factor 2 (KLF2) regulates proinflammatory activation of monocytes. Proceedings of the National Academy of Sciences of the United States of America.

[50] Mitri Z, Constantine T, O'Regan R (2012). The HER2 Receptor in Breast Cancer: Pathophysiology, Clinical Use, and New Advances in Therapy. Chemotherapy research and practice.

