# Supplementary material for: Kruppel-like factor 2 suppresses mammary carcinoma growth by regulating retinoic acid signaling
Source: Oncotarget. 2015 Sep 21;6(34):35830–42. doi: 10.18632/oncotarget.5767 (PMC4742144; doi:10.18632/oncotarget.5767)
Supplement: Supplementary file 1 [file oncotarget-06-35830-s001.pdf]

Kruppel-like factor 2 suppresses mammary carcinoma growth by regulating retinoic acid signaling

Supplementary Material

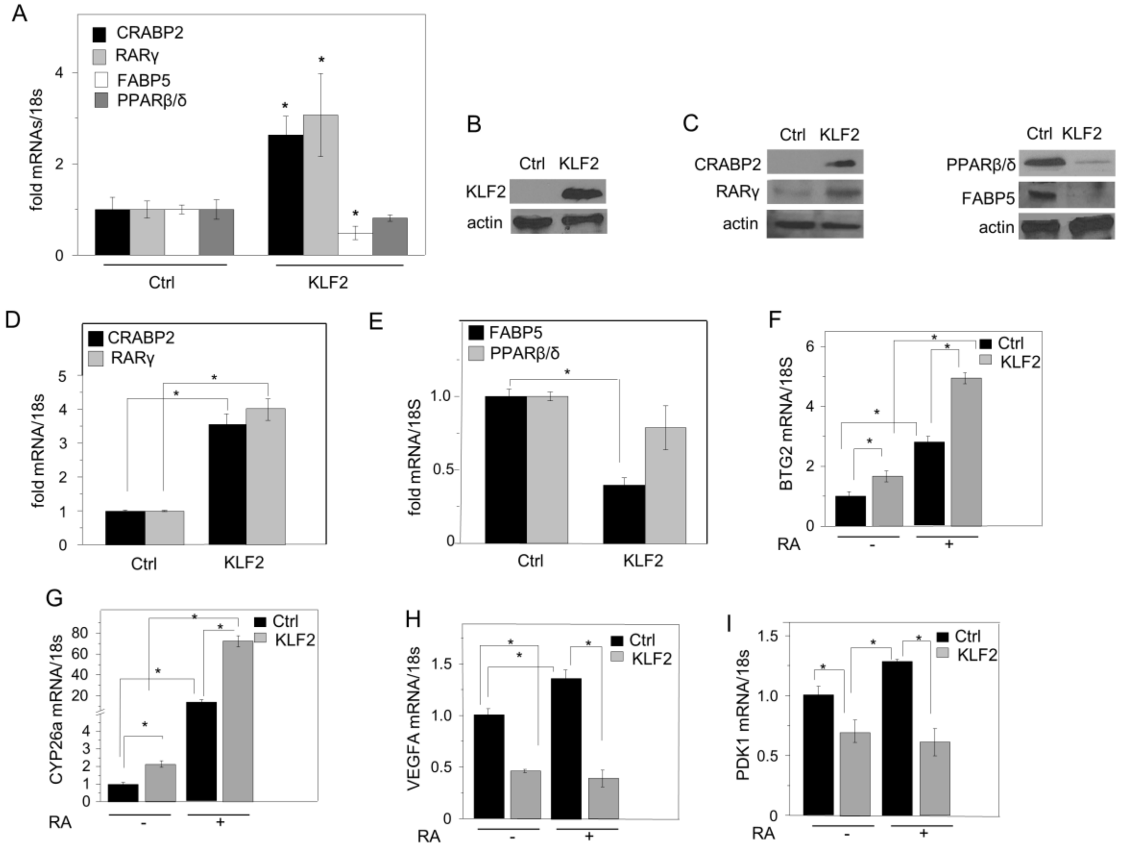

Figure S1 KLF2 upregulates CRABP2 and RARγ and downregulates FABP5 and PPARβ/δ in MDA-MB-231 cells. A) Levels of CRABP2, RARγ, FABP5 and PPARβ/δ mRNA in tumors that arose from parental and KLF2 overexpressing MDA-MB-231 cells, measured by Q-PCR. Mean±SD, n=3. *$p<0.05$, paired Student's T-test. B) Immunoblots demonstrating expression of KLF2 in MDA-MB-231 cells stably expressing GFP (Ctrl) or GFP-KLF2 (KLF2). C) Immunoblots demonstrating expression of CRABP2 and RARγ (left) and FABP5 and PPARβ/δ (right) in MDA-MB-231 cells stably expressing

GFP (Ctrl) or GFP-KLF2 (KLF2). D), E) Levels of CRABP2 and RARγ (D), FABP5 and PPARβ/δ (E) mRNA in GFP (Ctrl) and GFP-KLF2 (KLF2) overexpressing MDA-MB-231 cells Mean±SD, n=3. *p<0.05, paired Student's T-test. F)-I) MDA-MB-231 cells stably overexpressing GFP (Ctrl) or GFP-KLF2 (KLF2) were treated with vehicle or RA (1 μM) for 4 h. Levels of mRNA for BTG2 (F), CYP26a (G), VEGFA (H) and PDK1 (I) were measured by Q-PCR. Mean±SD, n=3. *p<0.05, paired Student's T-test.

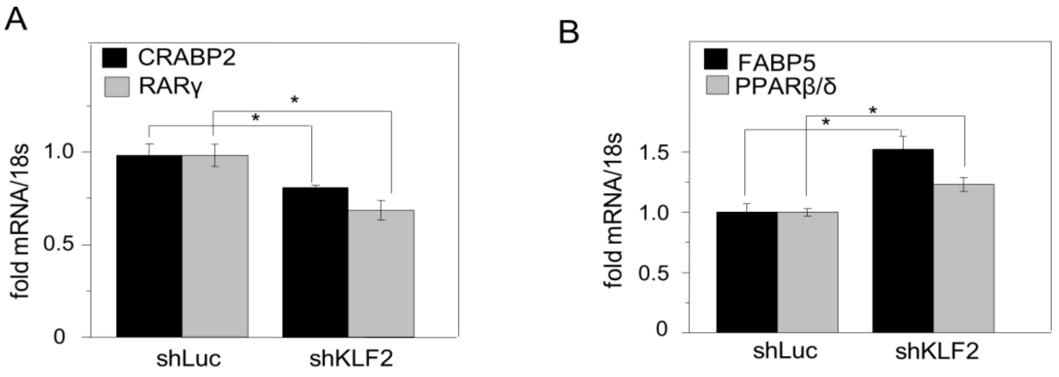

Figure S2 Downregulation of KLF2 suppresses CRABP2 and RARγ and upregulates FABP5 and PPARβ/δ mRNA in MCF-7 cells. A) Levels of CRABP2 and RARγ mRNA in MCF-7 cells expressing shLuc or shKLF2, measured by Q-PCR. Mean±SD, n=3. *$p<0.05$, paired Student's T-test. B) Levels of FABP5 and PPARβ/δ mRNA in MCF-7 cells expressing shLuc or shKLF2 were measured by Q-PCR. Mean±SD, n=3. *$p<0.05$, paired Student's T-test.

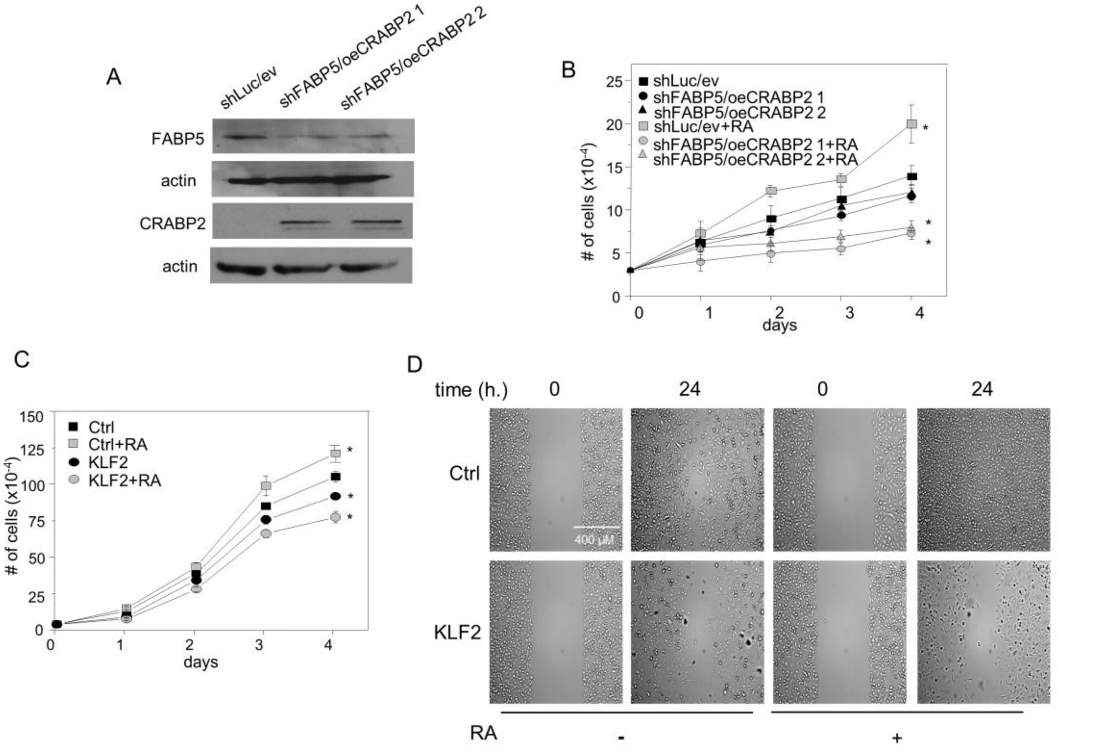

Figure S3 KLF2 regulates RA activities. A) Immunoblots demonstrating levels of FABP5 and CRABP2 in MDA-MB-231 cells transfected with a control vector harboring shLuc together with an empty control vector or vector encoding two different shFABP5 (shFABP5 1 or shFABP5) together with an expression vector harboring CRABP2. B) MDA-MB-231 cells were transfected with a control vector encoding shLuc together with an empty vector or with a vector harboring two different shFABP5 together with an expression vector encoding CRABP2. Cells were treated with ethanol or RA (1 µM) for 4 days. RA was replenished every 24 h. Cells were counted at the indicated time. Mean±SD, n=3. *p<0.05 by paired Student's T-test. C) MDA-MB-231 cells stably overexpressing GFP (Ctrl) or GFP-KLF2 (KLF2) were cultured in medium containing 10% charcoal-treated FBS and treated with ethanol or RA (1 μM) for 4 days. Cells were counted at indicated time. *p< 0.05 (n=3) by paired Student's T-test. D) Wound healing

assays using MDA-MB-231 cells stably overexpressing GFP (Ctrl) or GFP-KLF2 (KLF2) and treated with RA (1 μM) or vehicle for 24 hours. Images were obtained using ZEISS Fluorescent Microscopy.

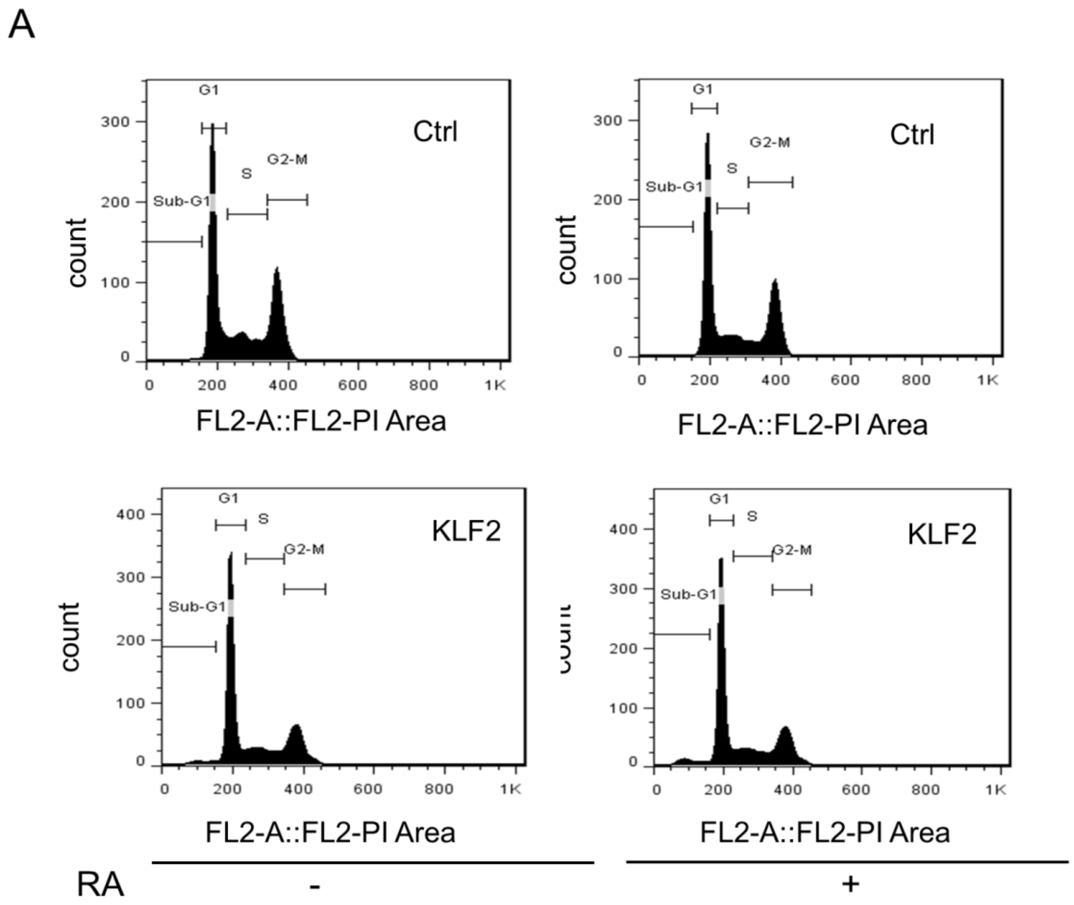

Figure S4 KLF2 enhances RA induced apoptosis on MDA-MB-231 cancer cells. A) MDA-MB-231 cells that stably overexpress GFP (Ctrl) or GFP-KLF2 (KLF2) were cultured in medium containing 10% charcoal-treated FBS in the absence or presence of RA (1 μM) for 5 days. RA was replenished every 24 h. Cells were harvested, washed with PBS, fixed in 70% ice cold ethanol, and stained with propidium iodide. Fractions of cells in different cell cycle stages were assessed by FACS.
